# Integrated Analysis of DNA Methylation and mRNA Expression Profiles to Identify Key Genes in Severe Oligozoospermia

**DOI:** 10.3389/fphys.2017.00261

**Published:** 2017-05-12

**Authors:** Zhiming Li, Xuan Zhuang, Jinxiong Zeng, Chi-Meng Tzeng

**Affiliations:** ^1^Translational Medicine Research Center, School of Pharmaceutical Sciences, Xiamen UniversityXiamen China; ^2^Department of Pathology, Wake Forest University School of MedicineWinston-Salem, NC, USA; ^3^Key Laboratory for Cancer T-Cell Theranostics and Clinical TranslationXiamen, China; ^4^Department of Urology, The First Affiliated Hospital of Xiamen UniversityXiamen, China; ^5^ChinaCredit Andrology Medical Co., Ltd.Shenzhen, China; ^6^INNOVA Cell Theranostics/Clinics and TRANSLA Health GroupYangzhou, China

**Keywords:** severe oligozoospermia, obstructive azoospermia, DNA methylation, mRNA expression, integrated analysis

## Abstract

Severe oligozoospermia (SO) is a complex disorder, whose etiology is the combined effect of genetic factors and epigenetic conditions. In this study, we examined DNA methylation and mRNA expression status in a set of testicular tissues of SO patients (*n* = 3), and compared methylated data with those derived from obstructive azoospermia (OA) patients (*n* = 3) with normal spermatogenesis phenotype. We identified 1,960 differentially methylated CpG sites showing significant alterations in SO vs. OA using the Illumina Infinium HumanMethylation450 bead array. By integrating above DNA methylation data and mRNA expression results, we totally identified 72 methylated CpG sites located in 65 genes with anti-correlation between DNA methylation and mRNA expression. Integrated pathways analysis indicates that these genes are involved in response to hormone stimulus, activation of protein kinase activity, and apoptotic process, among others. We also observed some genes with inversely correlated difference is novel in male infertility field, including PTPRN2, EPHX1, SERPINB9, SLIT3, etc. Our results lay a groundwork for further biological study of SO. Moreover, we generated a workflow for integrated analysis of DNA methylation and mRNA expression, which is expandable to other study types.

## Introduction

Fertility is a major worldwide health problem and affects about 15% of couples, 40–50% of which are attributed to male factors (Anderson et al., 2009). Genetic defects contribute to 15–30% cases of male infertility. Recently, great attentions has been paid to the epigenetic studies due to the great achievement on the novel knowledge in the regulation of gene expression. In contrast to the definition of genetic defects that modify the DNA sequence itself, epigenetic defects are heritable alterations in the function of specific genes but not related to modification in the DNA sequence (Stuppia et al., 2015). DNA methylation is one of the most extensively investigated epigenetic mechanisms. DNA methylation is the stable, covalent addition of a methylgroup to 5′- position of cytosine residues, mainly in CpG dinucleotides. In human, nearly 60–80% CpG are methylated in the promoter regions. Methylation of CpG within the promoter regions commonly leads to the silence of transcription process. In mammals, formation of DNA methylation pattern is one of the important epigenetic events that affect development of various tissues, including testis. The abnormal sperm DNA methylation patterns of both imprinted and non-imprinted genes are proved to be associated with infertility or subfertility in oligospermic men (Urdinguio et al., 2015). However, no previous studies of the global DNA methylation aberrations have been reported in severe oligozoospermia (SO).

SO (sperm concentration <5 million/ml) is found in around 5% of couples presenting with infertility. The pathogenesis of this disease remain largely unknown, although deletions of the Deleted in Azoospermia -like (DAZL) gene were demonstrated to be responsible for severe oligozoospermia in some extent (Fernandes et al., 2006). Moreover, due to the reported heredity of genetic defects, such as Y chromosome microdeletions, the importance of careful evaluation of associated genetic factors in male infertility prior to assisted reproduction is evident. Early studies performed in imprinted genes suggested that there was an enhanced risk of congenital imprinting disorders in children conceived through assisted reproductive technologies (ART). Aberrant DNA methylation patterns have also been proved in some non-imprinted genes involved in spermatogenesis impairment, such as MTHFR, CREM and DAZL (Urdinguio et al., 2015). It is well established that alterations of DNA methylation and differences of gene expression are causally related, with hypomethylation generally resulting in gene expression, and hypermethylation leading to gene silencing. The purpose of this study was to identify key gene determined by both regulatory mechanisms from the testicular tissues of infertile patients via integrated analysis, and to elucidate the underlying biological significance.

**Figure 1:**
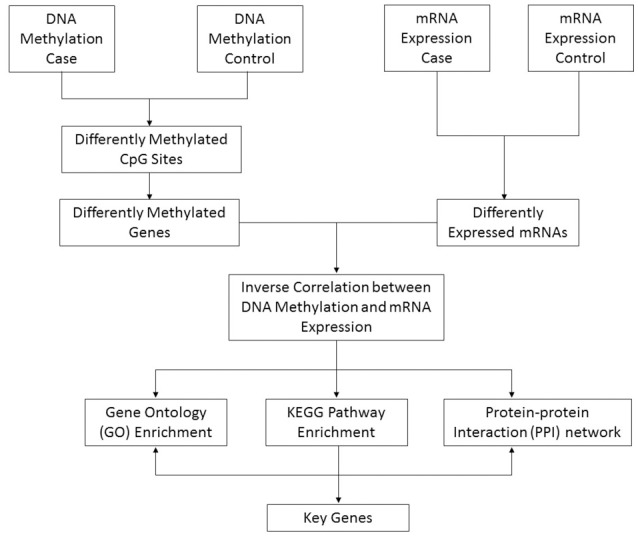
**Schematic pipeline depicting the strategy of identification of key genes from DNA methylation and mRNA expression array integrative data**. The method proceeds as follows steps: (1) Identify significantly differentially methylated genes and expressed genes in the case and control groups; (2) Retain those genes whose methylation and expression levels are highly anti-correlated; (3) Enrich the Gene Ontology, KEGG pathway, and PPI networks of anti-correlated genes; (4) Identify key genes from a combination of biological information, including interactions and relationships among genes, gene functional annotations and pathway maps.

By using an Illumina Infinium Human Methylation 450K BeadChip DNA methylation array and Agilent SurePrint G3 Human Gene Expression 8 × 60 K platform, we systematically defined DNA methylation and mRNA expression profiles in testicular tissues of SO (*n* = 3) by comparing obstructive azoospermia (OA) patients (*n* = 3) with normal spermatogenesis (included as controls). We considered that the list of differentiated genes contributes to tissue specific functions and integrated data improve us better understanding of the mechanisms of SO. In this article, moreover, we propose an approach to common human disease that incorporates DNA methylation and mRNA expression profiles (Figure 1).

## Materials and methods

### Patients

Testicular biopsy specimens were obtained from 3 patients (aged from 23 to 37 years) with SO and from 3 patients (aged from 25 to 28 years) with OA for the microarray analysis. These patients underwent testicular sperm extraction (TESE) for assisted reproduction and/or diagnostic biopsy for histological examination. For the histological evaluation, the specimens were stained with hematoxylin and eosin (H&E) and analyzed by microscopy. SO was defined as a reduced number of sperm in the male ejaculate or less than 5 million sperm per milliliter. OA was defined as in the reference (Okada et al., 2008): (1) motile spermatozoa were obtained from a microsurgical epididymal sperm aspiration (MESA) or (2) a great number of mature spermatozoa were provided by TESE. The ideal controls in the study would be the normal male population with known fertility, however, the difficulties in sampling testicular tissues leads to this strategy impractical. Instead, control samples of urology males who had no history of meiotic impairment or infertility and exhibited normal spermatogenesis upon histological examination. Karyotype analysis and Y-chromosome microdeletion analysis were performed on all patients to confirm a normal karyotype. Additionally, none of the controls experienced adjuvant hormonal treatment prior to orchiectomy. The infertile male patients who visited Xiamen University Affiliated First Hospital had a routine semen examination on the basis of 2010 WHO criteria. The ethics committees of Xiamen University Affiliated First Hospital (Institutional Review Board Number: KYX-2015-001) approved the study design. All subjects provided written informed consent.

### RNA extraction

Immediately after retrieval, the samples were stored at −80°C until further RNA process. Total RNAs (including miRNAs) from the frozen testicular tissues was extracted with the miRNeasy Micro Kit (Catalog no. 217084, Qiagen, Germany) following the manufacturer's protocol. On-column DNase digestion was performed to eliminate DNA contamination. The RNA concentration, purity, RNA integrity number (RIN) were determined using a NanoDrop ND-1000 spectrophotometer (Peqlab, Erlangen, Germany), an Agilent 2100 Bioanalyzer and an RNA 6000 NanoLabChip Kit (Agilent Technologies), respectively.

### Gene expression microarray

For microarray hybridization, 100 ng of total RNA were prepared using the Agilent's One-Color Microarray-Based Gene Expression Analysis Low Input Quick Amp Labeling kit (Agilent 5190-2305) according to the manufacturer's protocol, and were then hybridized to the Agilent SurePrint G3 Human gene expression 8 × 60 K microarray following the manufacturer's instruction. In short, an input of 100 ng of entire RNA was used to generate cDNAs, followed by *in vitro* transcription and incorporation of cyanine-3 into the nascent cRNAs. The cyanine-3-labeled cRNAs were fragmented and hybridized to the array for 17 h at 65°C in an Agilent hybridization oven. Arrays were scanned with the Agilent G2565CA microarray scanner at 5 μm resolution. The microarray data were processed with Agilent Feature Extraction 10.7.3.1 Software (Agilent Technologies).

### Gene expression microarray data analysis

The raw signal intensity data were normalized with GeneSpring GX software version 12.0 (Agilent Technologies). Specifically, the signal values were log 2 transformed using the quantile method (Bolstad et al., 2003). Unequal *t*-test (*p*-values) was applied to identify the differentially expressed genes in SO from OA. The *p*-values were corrected by false discovery rate (FDR) of Benjamini and Hochberg (*q*-values). Fold change (FC) values were calculated for each gene as the difference of the mean intensity of the SO samples from OA samples. Genes with the *FC* value > 2 or < 1/2 and the *q*-value < 0.05 were considered as differentially expressed. Quality control analysis of microarray gene expression data was performed as previously described (Zahurak et al., 2007).

### DNA isolation and preparation

Testicular tissues were dissolved in 200 μl lysis buffer of DNA Micro Kit (Catalog no. 56304, Qiagen, Germany), and incubated with proteinase K overnight at 56°C for two nights. DNA was extracted according to the manufacturer's protocol (QIAamp DNA Micro Kit, Qiagen), and DNA concentration was determined at 260 nm using the NanoDrop ND-1000 spectrophotometer (Nanodrop Technologies Inc., Wilmington, NC, USA). Bisulfite modification of 500 ng DNA of each samples was carried out with the EZ DNA methylation kit (Zymo Research, Orange, CA).

### DNA methylation microarray

Infinium HumanMethylation450 BeadChips (Illumina, Inc.) were used to analyze DNA methylation. With this analysis, it is possible to cover >485,000 methylation sites per sample at single nucleotide resolution. This BeadChip covers 99% of RefSeq genes with an average of 17 CpG sites per gene distributed across the promoter, 5′UTR, first exon, gene body, and 3′UTR regions (Bibikova et al., 2011). Additionally, it covers 96% of CpG islands, with an average of five CpG sites each, as well as the corresponding shores and shelves. After bisulfite conversion, we used 4 μl of bisulfite-converted DNA to hybridize on the array. A pair of bead-bound probes is applied to quantify the amount of thymine or cytosine in the process of hybridization. Fluorescent signal of two bead types are obtained from BeadArray Reader. The methylation status of a CpG site was calculated with the average beta-value, which was between 0 (unmethylated) and 1 (completely methylated). Beta-value is the ratio of the signal intensity of the methylated probe to the sum of the methylated and unmethylated probes.

### Detection of differentially methylated probes

The raw data were subject to quality control and normalization with the recommended guidelines of the bioconductor R package minfi. Wilcoxon rank test was performed to compare SO samples to OA samples. Probes with *p*-value was <0.05 and the delta beta (Δβ) >0.2 or <0.2 were considered statistically significant and differentially methylated. Delta beta value is defined as the average beta value of SO samples minus the average beta value of OA samples.

### Integrating gene expression and DNA methylation

To perform the integrated analysis of DNA methylation and gene expression, we used a two-step analysis process. (1) Identify differentially methylated genes in two groups. Genes differentially methylated between SO and OA groups were selected if the absolute value of delta beta was greater than 0.2 and *p*-value < 0.05. (2) For each differentially methylated gene, test whether there is a strong association between DNA methylated expression and transcriptional expression. Spearman rank correlation for the median of the methylation level of CpGs in on amplicon against the expression probes on the Agilent expression arrays was used to access their correlations. In this analysis, we applied correlation coefficient rho and *p*-value to investigate the correlation and significance between mRNA expression and DNA methylation with the function cor.test in R. Significantly negative correlation was considered if rho < 0 and *p*-value < 0.05, and significantly positive correlation was considered if rho > 0 and *p*-value < 0.05.

We next conducted unsupervised clustering analysis and displayed a different separation of SO and OA with evidence of gene clusters that are differentially expressed or methylated. Hierarchical clustering was performed using Ward linkage with Euclidean distance for samples. A clustering method available in Charm package was applied to the differentially methylated CpGs (Aryee et al., 2011).

### Gene Ontology (GO) and kyoto encyclopedia of genes and genomes (KEGG) analysis

To find biological mechanisms underlying the genes analyzed above, we conducted GO term and KEGG pathway analysis using Cytoscape V2.7 (http://cytoscape.org/) with the ClueGo V1.3 plug-in (Bindea et al., 2009), which is able to extract biological features and annotations of anti-correlated genes. To only obtain significant enrichment categories of GO terms and KEGG pathways, the *p*-value was performed with right-sided hypergeometric tests and corrected by Benjamini-Hochberg adjustment (*q*-value). The categories with *q*-value < 0.05 were selected for further analysis, which were considered to be statistically significant deviation from the expected distribution. Results were visualized with the ClueGO to generate clusters of functions associated with genes.

### Protein-protein interaction (PPI) network construction and analysis

The latest experimentally confirmed human PPI data is available from human protein-protein interactions database (HPRD) (http://www.hprd.org/), which has been widely applied in human PPI network research for various disease. NetworkAnalyzer (Assenov et al., 2008) (http://www.mpi-inf.mpg.de/) is used to analyze the topological properties of biological networks, describing networks as collections of nodes and edges. Cytoscape software was used for network visualization.

## Results

### Identification of differentially methylated regions in SO

**Figure 2:**
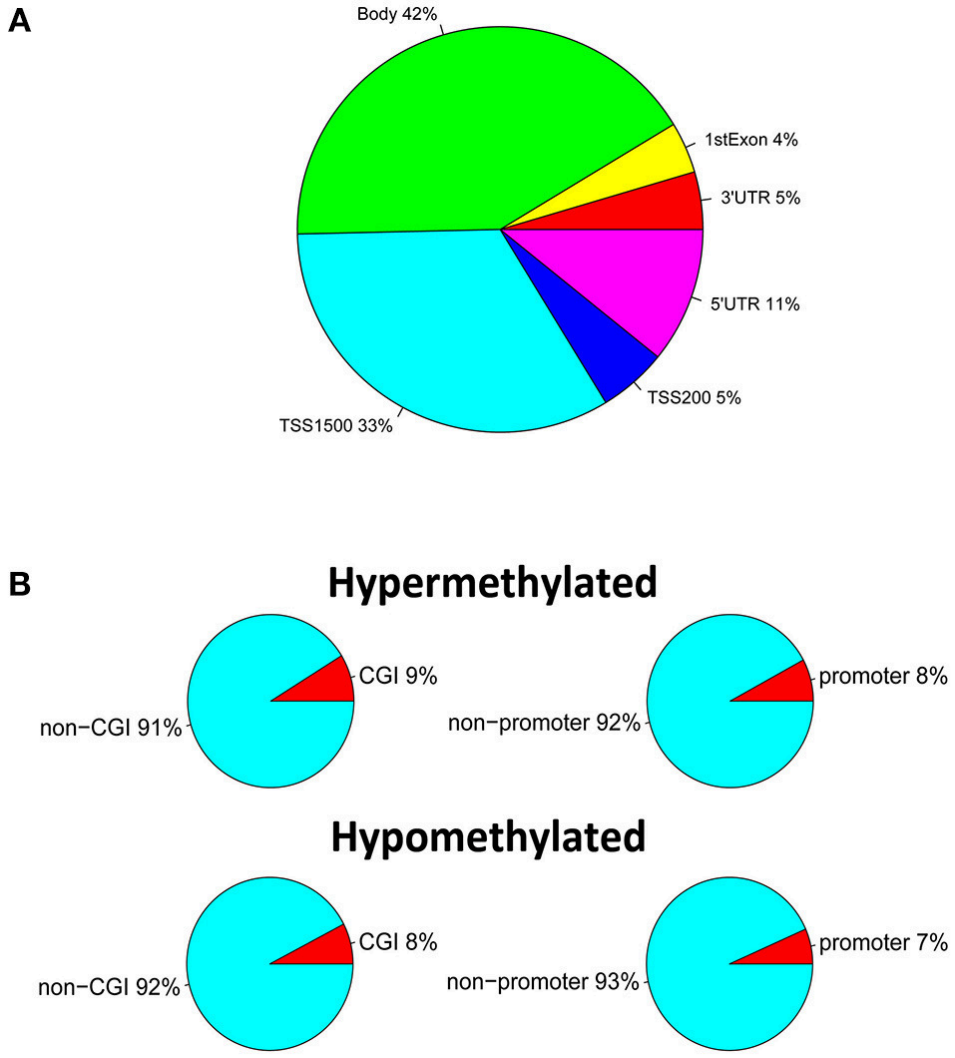
**Identification of DNA methylation differences between SO and OA. (A)** Genomic distribution of differentially methylated probes regarding their respective location to genes. **(B)** Proportions of hypermethylated and hypomethylated probes from genes with associated CpG islands (CGI) and probe locations, categorized as promoter (±1 kb from TSS) or non-promoter regions.

We performed high-throughput DNA methylation screening to compare testicular tissue-specific DNA patterns in three SO and three OA patients. To this end, we extracted genomic DNA from each sample, and made a treatment with sodium bisulfite, and calculate the DNA methylation level via the Illumina Infinium HumanMethylation450 BeadChip, which covers more than 450,000 CpG sites across the 99% of RefSeq genes. After filtering the raw data and statistical analysis, we determined that 1960 CpG sites, related to 1440 different genes, showing significant differences of DNA methylation in SO, compared with controls (absolute value of delta beta > 0.2, *p* < 0.05). Specifically, there were 197 hypomethylated CpG sites located in 149 genes and 1763 hypermethylated CpG sites associated with 1,317 genes. The majority of the 1,960 differentially methylated sites were located in gene bodies (42%) and within 1,500 bp from the transcription start site (TSS1500) (33%) (Figure 2A). Only 5% differentially methylated sites located in the 3′UTR region, or within 200 bp from the transcription start site (TSS200). There are 4 and 11% differentially methylated sites in first Exon and 5′UTR region, respectively. Moreover, based on the functional distribution standpoint, we also analyzed the amount of differentially methylated probes in CpG islands (CGI) and promoters of corresponding genes of SO vs. OA (Figure 2B). Fort the CGI distribution, 9% of CpGs in CGI was hypermethylated, vs. 8% of CpG in CGI was hypomethylated. Additionally, 8% of CpG in promoter regions (defined as the region 1 kb upstream to 1 kb downstream of the transcription start site) hypermethylated, vs. 7% of CpG in promoter was hypomethylated.

In the differentially methylated genes from the comparison between SO and OA include a number of genes with known functions in male infertility, and also some potentially novel genes for male infertility. SOX30 is one of the good examples of highly interesting genes. The SOX gene encodes a group of transcriptional factors containing a DNA binding HMG-box domain, which play important roles for the sex determination protein called SRY. In human and mouse studies, Sox30 is found to be associated with spermatogonial differentiation and spermatogenesis (Ballow et al., 2006). However, up to now, the precise function and regulatory mechanism of Sox30 have remained unknown. Recently, Han et al. found that Sox30 expression is under the control of DNA methylation status, and this expression pattern is associated with testis development in mice (Han et al., 2014). Our results indicated that SOX30 is hypermethylated in SO with respect to OA, and that hypermethylation is probably related to SOX30 down-expression in patients with SO. Another interesting example in the hypermethylated gene list is PTPRN2, a receptor-like protein tyrosine phosphatase that modulate plasma membrane PI(4,5)P2 levels to promote actin remodeling. We identified that PTPRN2 gene presented highly differentially methylated difference in SO vs. AO. PTPRN2 lately was reported to be near the hypermethylated regions of sperm DNA in the patients with failure-to-conceive (Jenkins et al., 2016). Conversely, some hypermethylated genes (such as, DENND2D and LOC401127) and hypomethylated genes (such as, HLA-DPA1 and COL18A1) have not previously been associated with male infertility, which may provide novel insight into the etiology of idiopathic SO.

### Integration of DNA methylation data with expression data

**Figure 3:**
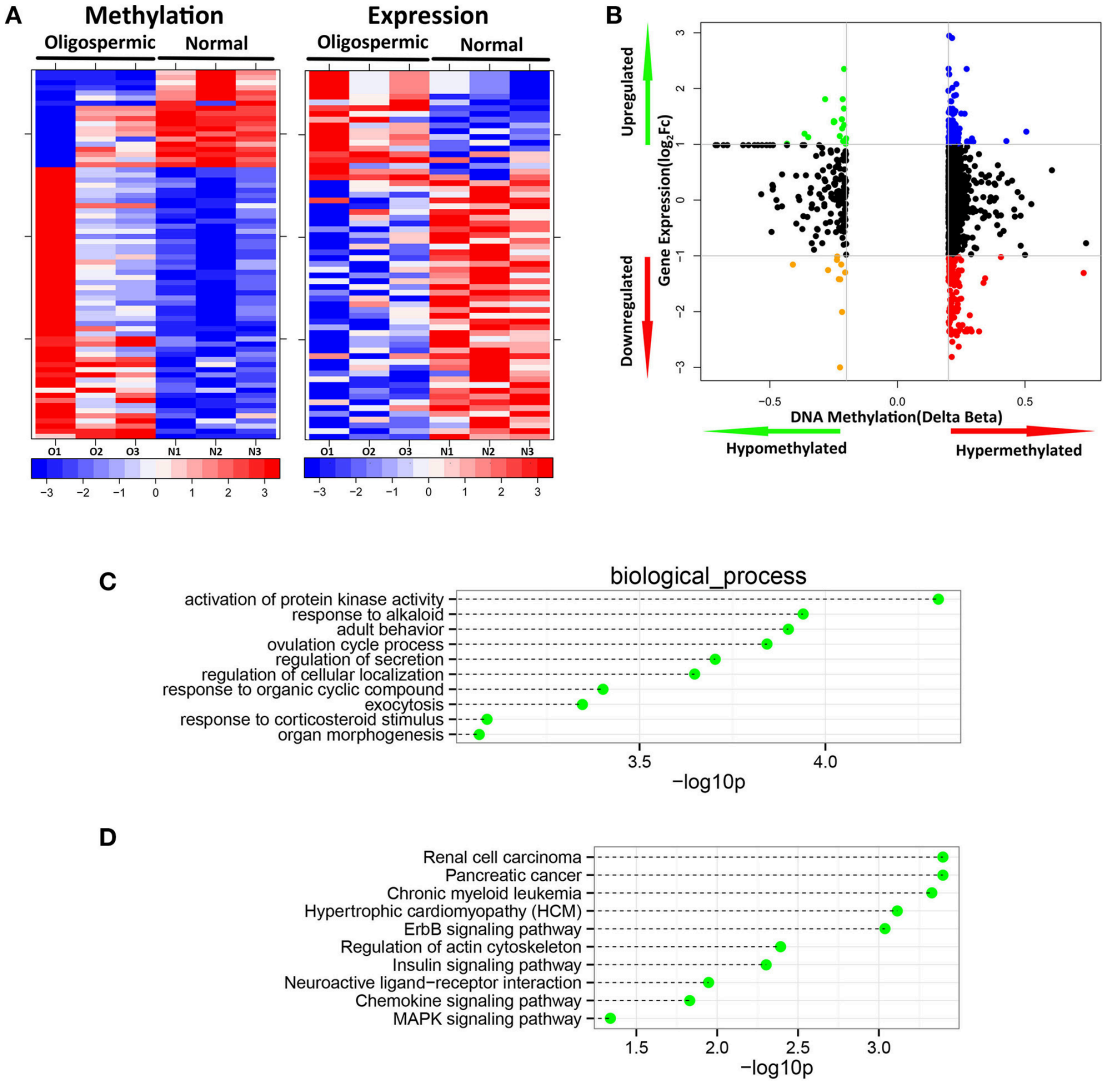
**Integration of DNA methylation with expression data. (A)** Heatmap comparison of inversely correlated expression and methylation. **(B)** Starburst plot integrating differential DNA methylation and gene expression analysis. Indicated are genes that are hypermethylated and down-regulated genes (red); hypomethylated and up-regulated genes (green); hypermethylated and up-regulated genes (blue); or hypomethylated and down-regulated genes (orange); **(C)** The top 10 most significantly enriched biological process categories; and **(D)** KEGG pathways within genes showing significant DNA methylation changes associated with significant inverse gene expression changes.

**Figure 4:**
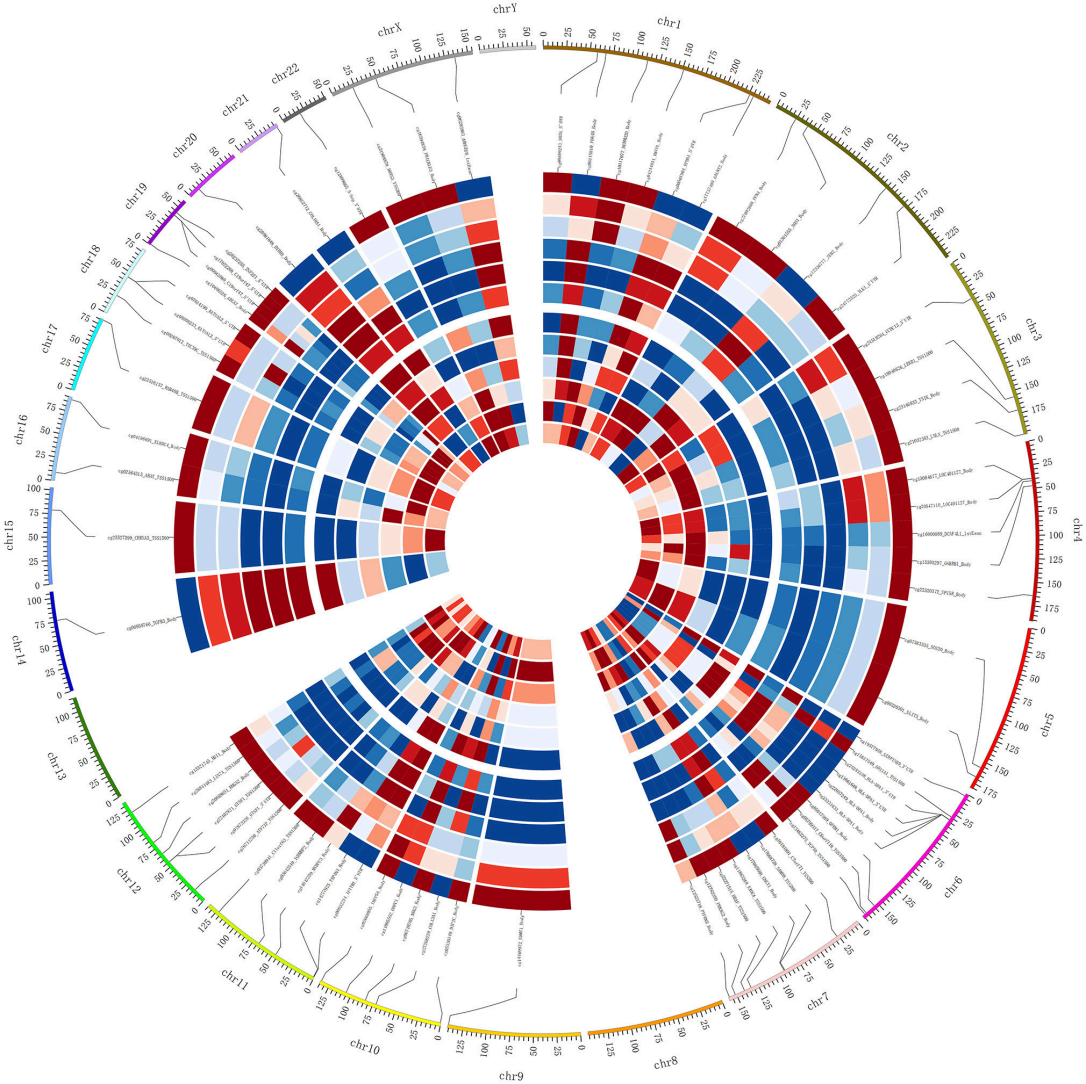
**Integrated Circos plot of genes showing coordinately inversed DNA methylation and gene expression**. The outermost circle displays the human chromosomes. The inner six circles (three SO and three OA samples displayed from outside to inside) represent the genome-wide distribution of differentially methylated probes. The innermost circles (three SO and three OA samples displayed from outside to inside) represent the genome-wide distribution of differentially expressed genes. The red indicates hypermethylation or overexpression, and blue indicates hypomethylation or under-expression.

DNA methylation usually is considered to repress the transcriptional expression, notably when methylated sites located at the CGI of promoters. To further study the implication of DNA methylated patterns in the regulation of gene expression, we compared the detected DNA methylated profiles with our gene expression data obtained from the same sample, which could contribute to reduce the influence of potential confounding factors. After Spearman rank, our integrated analysis showed that 72 annotated CpGs and 65 genes displayed an inverse correlation with coefficient rho < 0, *p*-value < 0.05 (Supplemental File 1). Table 1 shown the top 20 genes with the negative correlation of DNA methylation and mRNA expression of SO vs. OA. The two hierarchical clustering of the expression levels of just these 72 methylated probes and 65 genes could completely separate out SO and OA (Figure 3A). Of these, we identified 53 probes (located in 49 genes) that were significantly hypermethylated in SO, and 19 probes (located in 16 genes) that were significantly hypomethylated using our criteria of *p*-value < 0.05 and absolute delta beta > 0.2 (Figure 3B). The genomic locations of these 65 genes are visualized using Circos. Except for Chr8, Chr13, and ChrY, these genes were distributed in other 21 chromosomes. Clusters of hypermethylated and down-expressed genes were mainly located in Chr3, Chr7, and Chr9 (Figure 4). This global view when amplified is helpful for researchers to sinvestigate the detail relationship between chromosomes, gene expression, and DNA methylation.

**Table 1:** **Top 20 genes with the negative correlation of DNA methylation and mRNA expression between SO and OA**.

**Probe name**	**Region**	**Gene symbol**	**Gene description**	**Entrez gene**	***P*-value**	***Q*-value**	**Δβ value (SO-OA)**	**Fold change (SO/OA)**
cg13462275	TSS1500	TCP10	T-Complex 10	6953	0.000	0.000	0.263	0.572
cg07614190	5′UTR	KATNAL2	Katanin Catalytic Subunit A1 Like 2	83473	0.000	0.000	0.215	0.581
cg10117077	Body	DENND2D	DENN Domain Containing 2D	79961	0.002	0.080	0.483	0.567
cg24518264	5′UTR	CCDC13	Coiled-Coil Domain Containing 13	152206	0.002	0.080	0.250	0.473
cg08889243	5′UTR	DAB1	DAB1, Reelin Adaptor Protein	1600	0.002	0.080	0.247	0.892
cg07875328	5′UTR	GTSF1	Gametocyte Specific Factor 1	121355	0.002	0.080	0.238	0.562
cg14642259	Body	MYBPC3	Myosin Binding Protein C, Cardiac	4607	0.002	0.080	0.232	0.261
cg17072268	5′UTR	C19orf47	Chromosome 19 Open Reading Frame 47	126526	0.002	0.080	0.231	0.564
cg02668055	Body	INPP5A	Inositol Polyphosphate-5-Phosphatase A	3632	0.002	0.080	0.229	0.575
cg20847110	Body	LOC401127	WD Repeat Domain 5 Pseudogene	401127	0.002	0.080	0.226	0.597
cg22271515	TSS1500	BRAF	B-Raf Proto-Oncogene, Serine/Threonine Kinase	673	0.002	0.080	0.225	0.476
cg22520372	Body	NPY5R	Neuropeptide Y Receptor Y5	4889	0.002	0.080	0.215	0.609
cg03730941	TSS1500	C11orf63	Chromosome 11 Open Reading Frame 63	79864	0.002	0.080	0.205	0.657
cg00320301	Body	SLIT3	Slit Guidance Ligand 3	6586	0.002	0.080	0.204	0.520
cg27092869	Body	PFN4	Profilin Family Member 4	375189	0.002	0.080	0.204	0.552
cg20981848	Body	BTBD3	BTB Domain Containing 3	22903	0.002	0.080	−0.201	2.154
cg17668728	TSS200	SAMD9	Sterile Alpha Motif Domain Containing 9	54809	0.002	0.080	−0.209	2.544
cg11005552	Body	OBFC1	Oligonucleotide/Oligosaccharide Binding Fold Containing 1	79991	0.002	0.080	−0.237	1.652
cg19577958	3′UTR	SERPINB9	Serpin Family B Member 9	5272	0.002	0.080	−0.249	2.672
cg13523718	Body	PTPRN2	Protein Tyrosine Phosphatase, Receptor Type N2	5799	0.015	0.188	0.740	0.585

We then set out to determine these differentially methylated genes are involved in which biological significance relevant to SO pathogenesis. We therefore performed Gene Ontology analysis to test whether some molecular functions or biological processes were significantly associated with the genes shown the inverse difference in DNA methylation status between SO and OA. We detected some enriched GO terms showing significantly changes in SO (Figure 3C), including the following categories: activation of protein kinase activity (GO:0032147), response to alkaloid (GO:0043279), adult behavior (GO:0030534), ovulation cycle process (GO:0022602), regulation of secretion (GO:0051046), regulation of cellular localization (GO:0060341), response to organic cyclic compound (GO:0014070), exocytosis (GO:0006887), response to corticosteroid stimulus (GO:0031960), organ morphogenesis (GO:0009887). One of the hypermethylated but down-expressed genes, BRAF, plays important roles in the most significant biological process of the enrichment: activation of protein kinase activity (GO:0032147). Moreover, we also analyzed the significant pathways enriched by these 65 genes with inversely correlation between DNA methylation and mRNA expression. Signaling pathway analysis revealed functions in the regulation of actin cytoskeleton, ErbB signaling pathway, Insulin signaling pathway, neuroactive ligand-receptor interaction, chemokine signaling pathway, MAPK signaling pathway (Figure 3D). It has been previously reported that the intracellular pathways associated with spermatogenesis were subject to be modulated or damaged in male infertility, including MAPK signal transduction. The MAPK signaling pathway is a vital cellular signal cascade for the regulation of cell proliferation and differentiation for mammalian, which utilizes a chain of protein kinases to transduce signals from the receptors on the cell surface to the DNA in the nucleus. One gene of the series of molecules, BRAF, known to be one of components of MAPK signaling pathway, was down-expressed in mRNA level and hypermethylated in our platform. Interestingly, it has been demonstrated that BRAF-AKAP9 gene fusion leads to activate the MAPK pathway in human thyroid cancer (Ciampi et al., 2005), and AKAP9 is crucial for sertoli cell maturation and spermatogenesis in mice (Schimenti et al., 2013). This generated the hypothesis that MAPK dysregulation contributes to male subfertility might result from abnormal level of BRAF-AKAP9 gene fusion.

### Correlating network topology with SO mechanisms

**Figure 5:**
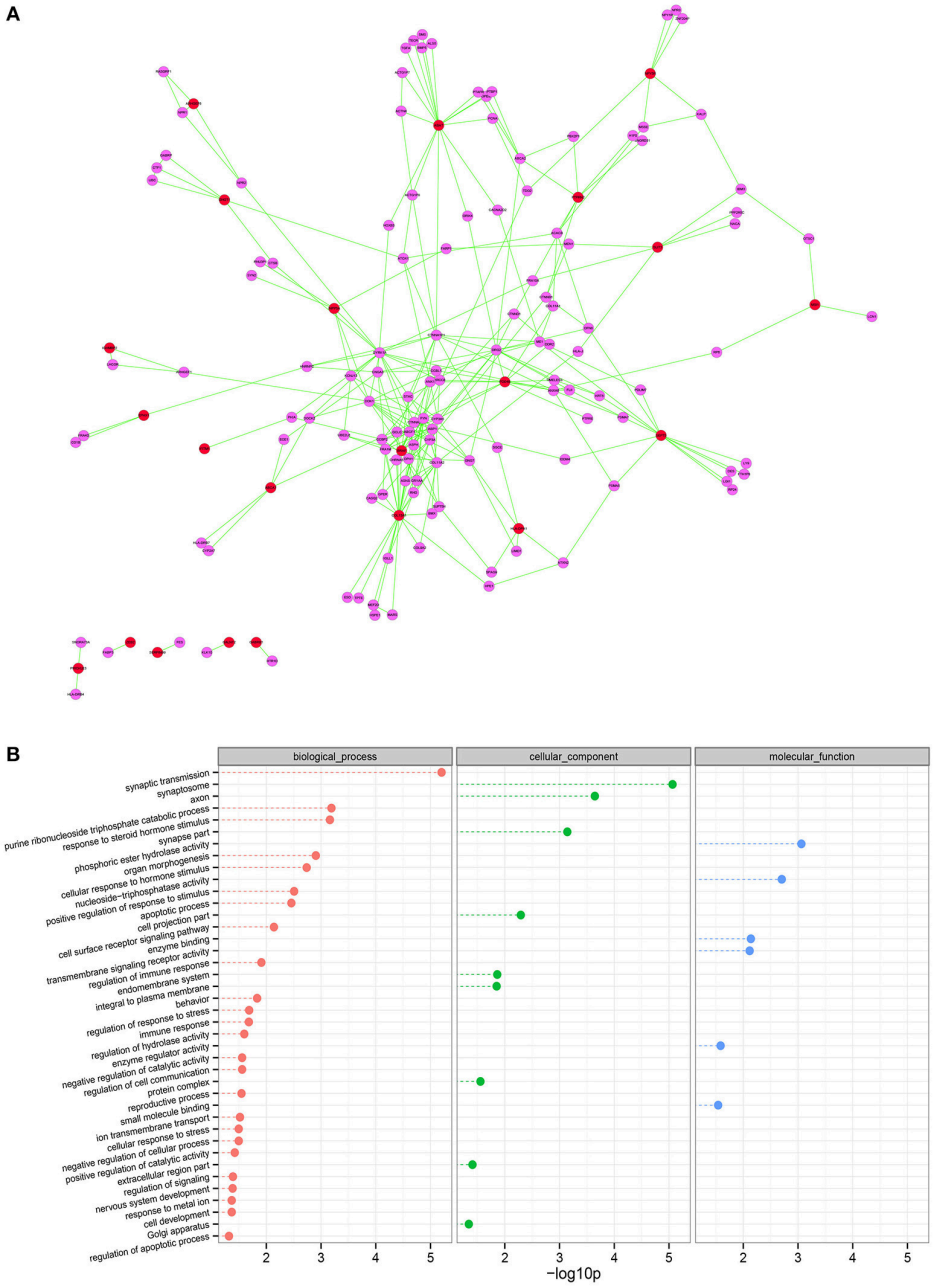
**Using network topology to infer elements involved in SO. (A)** Interaction network for genes differentially expressed and DNA methylated in SO. The red circles represent the hub genes inversely correlated DNA methylation and gene expression. The purple circles indicate the genes closely interacted with the hub genes that are highly linked nodes in network. **(B)** Gene ontology analysis of the relationship between connectivity of the genes in this network. Node shown in red, green, or blue is associated with biological process, cellular component, or molecular function, respectively. The *p*-values were calculated using hypergeometric tests and corrected using the Benjamini-Hochberg adjustment (*q*-value). The *q*-values are expressed and presented as negative logarithms (base 10).

We next examined the PPI subnetwork of 65 inversely correlated genes between mRNA expression and DNA methylation levels. The PPI network includes 148 nodes and 285 edges (Figure 5A). In the PPI network the nodes with high degree are defined as hub protein, and degrees are defined to measure how many neighbors a node directly connects to. Hub proteins in the network were considered to be the key nodes that regulate molecular mechanisms of the disease. In our PPI network, the top 10 hub proteins with significant degrees contained COL13A1 (degree, 23), FYN (degree, 18), ABAT (degree, 16), etc. (Table 2). For example, FYN, a kinase which is abundantly expressed in the testis and is involved in testis and sperm function, is closely associated with proper shaping of the head and acrosome (Luo et al., 2012). Furthermore, to identify the most relevant cellular activities controlled by these 22 both inversely correlated and PPI existed genes, we analyzed GO biological process terms and KEGG pathways. The most significant GO terms were related to synaptic transmission, purine ribonucleoside triphosphate catabolic process, response to steroid hormone stimulus, cellular response to hormone stimulus, positive regulation of response to stimulus, apoptotic process, cell surface receptor signaling pathway, regulation of immune response, behavior (Figure 5B). Three genes EPHX1, SERPINB9, and SLIT3 play common roles in the cellular response to the hormone stimulus (*q*-value = 0.001836) and the response to steroid hormone stimulus (*q*-value = 0.000691). Compared our data with those reported studies, we found that EPHX1 (Epoxide Hydrolase 1) appeared to be luteinizing hormone (LH) dependent in adult-type Leydig cells of mice (Griffin et al., 2010). In addition, genetic mutations in epoxide hydrolases affect the predisposition for oligozoospermia and asthenospermia in Han-Chinese population (Qin et al., 2012). SERPINB9, which hypopemethylation is associated with mRNA overexpression in our platform, probably protect against inadvertent release of granzyme B, and premature or unwanted activation of cell death pathways (Bird et al., 1998). Recently, Ottesen and his colleagues shown that SLIT3 is one of most strongly upregulated genes in the a spinal muscular atrophy model, which presented disturbed testis development, degenerated seminiferous tubules, decreased sperm count and low fertility (Ottesen et al., 2016). Several lines of evidence have shown that these key genes in our list are potentially involved in spermatogenesis, and may have clinical applications as novel biomarkers of male infertility.

**Table 2:** **PPI interaction network topological properties**.

**Protein ID**	**Protein name**	**Degree**	**Betweenness**	**Clustering coefficient**
1305	COL13A1	23	0.131843	0.083004
2534	FYN	18	0.144792	0.143791
18	ABAT	16	0.135937	0.025
1859	DYRK1A	16	0.2378	0.1
19	DRG2	15	0.114052	0.07619
1796	DOK1	13	0.0748	0.192308
5142	PDE4B	12	0.059437	0.136364
5413	SEPT5	12	0.085618	0.015152
1302	COL11A2	12	0.047586	0.227273
1496	CTNNA2	12	0.083287	0.121212

## Discussion

In this study, we have identified both novel and reported dysregulated genes involved in SO at the DNA methylation level and mRNA expression level. By using the integrated analysis of the DNA methylation and mRNA expression data, we have established a pipeline for investigating the complexity of gene dysregulation in the context of SO when using primary samples. The stepwise procedure is as follows: (1) Identify significantly differentially methylated genes and expressed genes in the case and control groups; (2) Retain those genes whose methylation and expression levels are highly anti-correlated; (3) Enrich the Gene Ontology, KEGG pathway and PPI networks of anti-correlated genes; (4) Identify key genes from a combination of biological information, including interactions and relationships among genes, gene functional annotations and pathway maps. In particular, the topological properties of this approach could be applied into explaining roles of unannotated proteins in diseases. The function of unannotated proteins could be predicted by further analysis of its direct neighbors and their neighborhood in the networks.

Recently, integrated analysis between transcriptional expression and DNA methylation has been carried out in a number of different disorders, including pilocytic astrocytomas (Zhou and Man, 2016), lung adenocarcinoma (Selamat et al., 2012), melanoma (Li et al., 2013), Crohn's disease-associated fibrosis (Sadler et al., 2016). For severe oligozoospermia, few studies have previously examined the existence of genes with aberrant DNA methylation or altered mRNA expression. There was no previous systematic effort to decipher the genetic mechanisms of SO in both regulatory layers. To our knowledge this study presents the first work of integrating microarray data of SO and OA in the progress of deciphering the cause of human male infertility. Our integrated analysis not only has allowed us to confirm changes (such as, PTPRN2 and FYN) described by others, but also to identify several novel genes with aberrant DNA methylation profiles, including DENND2D, HLA-DPA1, COL18A1. We also confirmed some genes that are relevant to the SO phenotype in mice model, such as SOX30 and EPHX1 (Han et al., 2014). Additionally, a small percent of methylation modifications in our SO result occurs at CGI of promoters and TSS downstream of genes. We observed that transcriptional activity is also affected by methylation alterations at gene bodies, which is corresponding to recent reports (Yang et al., 2014). We listed all above genes whose DNA methylation changes were inversely correlated with mRNA expression changes in the Supplemental Material, we also identified several genes that were coordinately hypermethylated and up-regulated, or hypomethylated and down regulated. While these two groups of genes do not fit into the classical paradigm of DNA methylation regulation, emerging evidence from recent high through-put studies show that DNA methylation regulation may be more complex. From other aspects, miRNA regulation is one of mechanisms also affects gene expressions (Zhuang et al., 2015; Li et al., 2016). Integration of the analysis of three profiles of mRNA expression, DNA methylation and miRNA expression suggests that the latter two regulations could act in the same or in opposite directions on gene expression (De La Rica et al., 2013).

In this study, a mixture of several kinds of cell was performed. Given that variations in the expression and methylation profiles might be affected by the difference in the ratio of each kind of testicular cell in the primary sample. Germ cell fractionation or laser microdissection also constitutes an approach of validating our present result. However, above two methods have their own limitations. Microdissection is time consuming for a large number of cells required in the microarray studies (Ferfouri et al., 2013). Germ cells fractionation seems impossible because a few amount of germ cells in the specimens of the SO patients. We can expect that much fewer cells will be required with the development of technology in the future.

The emergence of new technologies to analyze gene expression and DNA methylation has allowed the observation of changes at the global genome level. The primary sample is usually a good option for the research on male infertility using these high-throughput technologies. Distinct with many studies of integration analysis which used different resources of samples from the datasets, our study systematically investigated both DNA methylation and mRNA expression in order to overcome the limitations of sample size. In fact, integrative DNA methylation and gene expression analysis has been successfully used to identify a novel dysregulated biomarker discoidin domain receptor 1 (DDR1) in non-obstructive azoospermia (NOA) patients (Ramasamy et al., 2014). Given the small sample size and high-throughput data gathering techniques, the results should nevertheless be interpreted with caution. In our study, patients of two groups are selected for their similar pathological traits, including histology characteristics, hormone levels, and biochemical parameters (Supplemental File 2). Thus, the screened genes with significantly different expressions in subjects of two groups should be due to distinct pathology. Although these genes had statistically significant changes and are anti-correlated at the expression and methylation levels, the biologic significance remains unclear and further study is warranted. We believe our results may have potential implications in the diagnosis and therapy of spermotogenetic failure in the near years.

## Conclusion

This is the first integrated study of DNA methylation and mRNA expression of testicular tissues from SO and OA patients in order to effectively identify key genes underlying spermatogenesis processes and elucidate the biological mechanisms of SO. We systematically screened some genes with anticorrelation in two regulatory ways, which opens up many possibilities for epigenetic studies of male infertility. In addition, the approach described in our study provides a workflow to other researchers for exploring the concerned transcription dysregulated by DNA methylation.

## Author contributions

XZ collected clinical data and signed the testing report of each patient. ZL drafted the manuscript and analyzed the data. JZ designed the tables and figures. CT conceived the study and revised the manuscript.

### Conflict of interest statement

CT and JZ were employed by company INNOVA Cell Theranostics/Clinics and TRANSLA Health Group and ChinaCredit Andrology (Shenzhen) Medical Co., Ltd., respectively. The role of CT was study design, and JZ is responsible for language improvement. The other authors declare that the research was conducted in the absence of any commercial or financial relationships that could be construed as a potential conflict of interest.
