# Supplementary material for: Integrated Analysis of DNA Methylation and mRNA Expression Profiles to Identify Key Genes in Severe Oligozoospermia
Source: Front Physiol. 2017 May 12;8:261. doi: 10.3389/fphys.2017.00261 (PMC5427114; doi:10.3389/fphys.2017.00261)
Supplement: Supplementary file 2 [file Image1.PDF]

Supplemental figure 1. Sample histology characteristics. Photo-histogram from two representative testicular biopsies showing seminiferous tubules filled with disorganized abundant germ cells of obstructive azoospermia (OA) and incomplete spermatogenesis of Severe oligozoospermia (SO). Seminiferous tubules of SO have thickened basement membrane and hyperplastic Sertoli cells and Leydig cells. The spermatogenetic cells of different stages show moderate decrease, especially mature spermatid. There is some distribution of collapsed tubules. Seminiferous tubules of OA with evidence of spermatogenesis. Uniform distribution of non-collapsed tubules with normal appearance.

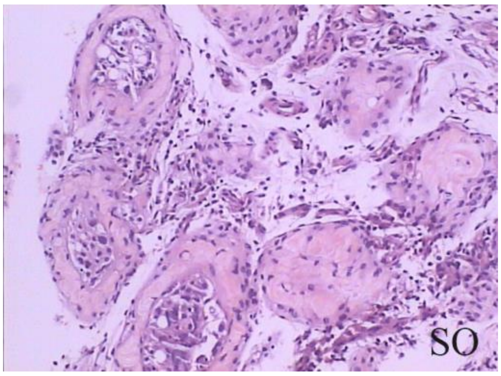

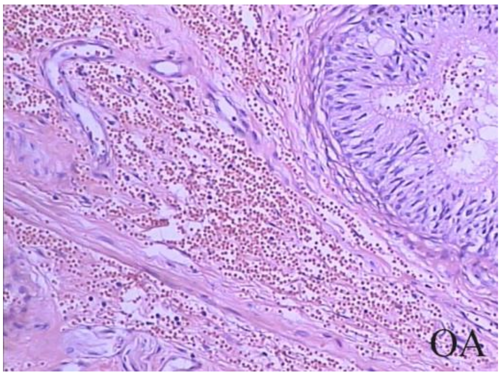

Supplemental table 1. Patient hormone and semen information

Group	No.	Sperm found via testicular extraction		FSH (IU/L)	LH (IU/L)	Testosterone (ng/dL)	Elastase (ng/mL)	Neutral α-glucosidase (mU/mL)	Fructose (mg/dL)
		Mature	Mobile						
SO	1	Yes	Yes	12.78	14.32	400.42	654.27	13.11	146.72
	2	Yes	Yes	13.12	15.87	419.98	578.54	15.28	145.29
	3	Yes	Yes	13.90	15.63	411.12	593.54	14.73	156.17
OA	1	Yes	Yes	8.23	9.76	399.21	178.79	25.67	289.35
	2	Yes	Yes	8.56	9.45	399.19	173.23	26.45	280.43
	3	Yes	Yes	7.23	9.98	400.78	188.31	29.90	290.48

FSH: follicle-stimulating hormone, LH：luteinizing hormone.
